# Daytime/nighttime levels of serum IL-33 in schizophrenia at hospital admission and before discharge

**DOI:** 10.1192/j.eurpsy.2023.2255

**Published:** 2023-07-19

**Authors:** J. J. Tascon-Cervera, A. Morera-Fumero, P. Abreu-Gonzalez, E. Diaz-Mesa, M. R. Cejas-Mendez, S. Yelmo-Cruz, L. Fernandez-Lopez, A. Marcos-Rodrigo

**Affiliations:** 1 Psychiatry, Universitary Hospital of Canary Island; 2 Departamento de Medicina Interna, Dermatología y Psiquiatría, Facultad de Ciencias de la Salud; 3 Ciencias Medicas Basicas: Unidad de Fisiologia, Facultad de Ciencias de la Salud, Universidad de La Laguna, San Cristóbal de La Laguna; 4 Psychiatry, Hospital Universitario Nuestra Señora de la Candelaria, Santa Cruz de Tenerife, Spain

## Abstract

**Introduction:**

It has been reported an inflammatory state in schizophrenia, with altered levels of some cytokines (Zhou et al. Cytokine 2021; 141:155441). Recent publications have shown the importance of IL- 33, a member of the IL-1 cytokine family which acts as an alarmin (Han et al. Neurosci Bull 2011; 27, 351-357). The role of this cytokine as a biomarker has been investigated in schizophrenia (Koricanac et al. Front Psychiatry 2022; 13, 925757). However, results are controversial. Some studies have not found significant associations between IL-33 and chronic schizophrenia (Campos-Carli et al. Compr Psychiatry 2017; 74 96-101), while other papers have reported increased levels (Kozlowska et. al. J Psychiatr Res. 2021; 138 380-387). In all these studies, levels of IL-33 were measured in a single daily measure, so that it has not been studied if IL-33 has changes during hospitalization.

**Objectives:**

To study the serum level of IL-33 at 12:00 and 00:00 hours in schizophrenia patients at admission and before hospital discharge.

**Methods:**

Fifteen inpatients with diagnosis of paranoid schizophrenia according to ICD-10 criteria were studied. Patients were hospitalized at the University Hospital of the Canary Islands psychiatric ward because of an acute relapse. A total of four blood samples were taken from each patient: at 12:00 and 00:00 hours the day after admission and at 12:00 and 00:00 hours the day before discharge. Serum IL-33 levels were measured by ELISA techniques. Daytime and nighttime IL-33 serum levels at admission and discharge were compared using a non-parametric Wilcoxon signed-rank test.

**Results:**

In table 1 the results of the comparison of IL-33 at admission and discharge are presented. There is a significant reduction of IL-33 levels at 00:00 h. at discharge in comparison with the IL-33 levels at 00:00 h. at admission (p=0.028). No other statistically significant differences were observed.

**Table:** 

Serum IL-33	Admission Mean±sd	Discharge Mean±sd	Z	P value
12:00 h.	191.0±348.7	247.0±378.2	-0.166	0.868
00:00 h.	218.8±370.3	153.6±275.7	-2.203	0.028

**Conclusions:**

The decrease of serum IL-33 at 00:00 at discharge compared to the 00:00 IL-33 serum level at admission points to the utility of this biomarker as a surrogate of brain inflammation.

**Disclosure of Interest:**

None Declared

